# No Health Without Brain Health & the Brain Health Mission: Raising Awareness & Putting the Brain on the Top of the Policy Agenda

**DOI:** 10.1192/j.eurpsy.2025.78

**Published:** 2025-08-26

**Authors:** S. Dickson, The European Brain Council

**Affiliations:** The University of Gothenburg, Gothenburg, Sweden

## Abstract

**Abstract:**

Brain health impacts everyone, in Europe and beyond. Whether it is the continued quest for cures and treatment for those living with brain conditions or the protection and fostering of healthy brains for our future generations, the challenges are unprecedented. To elevate brain health on the national, EU, and international policy agendas while making the most of existing initiatives, the European Brain Council (EBC) kicked off the No Health Without Brain Health campaign in the European Parliament during the 2024 Brain Awareness Week ahead of the 2024 European Elections.

This campaign is structured around two priority policy asks: the creation of a European Parliament Intergroup on Brain Health and Research and increased support to the creation of EU and National Brain plans. One year after the official kick-off, the campaign demonstrated that, when united, the brain community can reach a significant number of policymakers and make its voice heard to drive tangible policy changes.

The presentation “No Health Without Brain Health: Prioritising Brain Health in the European Union to Leave No One Behind” in this joint workshop will showcase success stories, address challenges and share good practices in EU-wide brain health initiatives. It will highlight the importance of collaboration and breaking down silos in preparing for the brain-healthy transition of our societies. Additionally, it will address the burden of brain disorders, neurological and mental alike, in a comprehensive and collaborative manner.

**Disclosure of Interest:**

None Declared

